# Scale-Up of a Newborn Resuscitation Capacity-Building and Skill Retention Program Associated With Improved Neonatal Outcomes in Gandaki Province, Nepal

**DOI:** 10.9745/GHSP-D-22-00046

**Published:** 2023-02-28

**Authors:** Robert B. Clark, Mala Chalise, Michael K. Visick, Vivek Ghosh, Ranjan Dhungana

**Affiliations:** a Brigham Young University, Provo, UT, USA.; b Children’s Medical Mission, Kathmandu, Nepal.; c School of Medicine, University of Utah, Salt Lake City, UT, USA.; d Safa Sunaulo Nepal, Kathmandu, Nepal.

## Abstract

**Background:**

Intrapartum events leading to asphyxia at birth are a leading cause of neonatal morbidity and mortality in Nepal. In response, the Nepal Ministry of Health and Population adopted the Helping Babies Breathe (HBB) training curriculum in 2015 as a tool to improve neonatal resuscitation and outcomes. Although the effectiveness of HBB training has been well documented, challenges remain in maintaining skills over time. Safa Sunaulo Nepal (SSN) designed an evidence-based intervention for scaling up newborn resuscitation training and skill retention. We report on its implementation and the changes in newborn outcomes during the program period.

**Methods:**

The program empowered facility-based trainers in newborn resuscitation and skill retention at 12 facilities in Gandaki Province. Seven of 14 level I hospitals and 5 of 6 level II hospitals were selected. A single external mentor coached the facility-based trainers, provided general support, and monitored progress. Program evaluation tracked changes in newborn metrics over 21 Nepali months (March 2018–November 2019). All deliveries occurring in the health facilities during the program period were included in the evaluation. We assessed program effectiveness by analyzing time trends of neonatal mortality, morbidity, and stillbirths.

**Results:**

We gathered data on neonatal health outcomes of 33,417 deliveries, including 23,820 vaginal deliveries and 9,597 cesarean deliveries. During the program, 43 facility-based trainers taught resuscitation skills to 425 medical personnel and supported skill retention. Neonatal deaths within 24 hours of birth (incidence rate ratio [IRR]=0.993, *P*=.044) and newborn morbidities (IRR=0.996, *P*<.001) showed a significantly declining trend.

**Conclusion:**

Our findings suggest that the SSN program had a substantial influence on critical neonatal outcomes. Future neonatal resuscitation capacity-building and skill retention efforts may benefit from incorporating elements of the program.

## Abstract (precis)

A program in Nepal based on the Helping Babies Breathe training curriculum successfully scaled up newborn resuscitation training and skill retention and could serve as an evidence-based model in other resource-constrained settings.

## Teaser

**Key Findings:**

- The Safa Sunaulo Nepal (SSN) program to scale up newborn resuscitation capacity-building and skill retention was associated with decreased neonatal death within 24 hours and decreased sick newborns discharged from the maternity unit.
- The program resulted in 43 facility-based trainers cascading resuscitation skills to 425 medical personnel and supporting skill retention over time.

**Key Implication:**

- Integrating elements of the SSN program—including a facility-based training cascade using the Helping Babies Breathe curriculum followed by practice and mentorship—may improve newborn resuscitation training and skill retention over time and subsequently improve neonatal outcomes.

## INTRODUCTION

Globally, approximately 2.4 million neonates die each year within the first 28 days of life, with intrapartum-related complications (perinatal asphyxia) as one of the major causes.^1^ Nearly 98% of neonatal deaths occur in low-resource countries, where 50% of the deaths occur on the day of birth.^2^ The worldwide mortality for children aged younger than 5 years has significantly decreased since 2000.^3^ However, reductions in neonatal mortality rates have lagged, leading to an increased proportion of overall child deaths attributed to the first month of life. For instance, 37% of the estimated child deaths in 1990 were attributable to neonatal deaths,^4^ compared with 47% in 2020.^1^ The UN Inter-agency Group for Child Mortality Estimation predicts that neonatal deaths will account for nearly 52% of child deaths by 2030.^1^ In light of continuing and unacceptably high rates of neonatal mortality in many low-resource countries post-Millennium Development Goals, the leaders of all United Nations countries committed to the Sustainable Development Goals, with the aim of Goal 3.2 to reduce neonatal mortality globally to 12 per 1,000 live births by 2030.^5^

In Nepal, significant improvement has been made in reducing the mortality rate for children aged younger than 5 years,^6^ which decreased from 133 deaths per 1,000 live births in 1991 to 29 deaths per 1,000 live births in 2016. However, neonatal mortality only dropped from 50 deaths per 1,000 live births in 1991 to 21 in 2016.^7^ Intrapartum events leading to asphyxia at birth continue to be one of the leading causes of neonatal morbidity and mortality in low-income countries, including Nepal.^8^^–^^10^

The Nepal Ministry of Health and Population (MOHP) has initiated a series of policies and programs to improve neonatal resuscitation in response to perinatal asphyxia. The Helping Babies Breathe (HBB) training curriculum,^11^ produced by the American Academy of Pediatrics, was introduced to physicians, midwives, hospital leaders, and the MOHP in 2012, followed by the initiation of training courses.^12^ In 2015, an abbreviated form of the HBB curriculum was included in the Skilled Birth Attendant In-service Training Package and the Community-Based Integrated Management of Childhood Illness Training Package countrywide. This inclusion was based upon, in part, the very successful pilot of HBB in Nepal in 2013.^13^ The dissemination of improved resuscitation techniques using HBB has been supported by multiple child health partners in Nepal.^14^^,^^15^ The National Health Training Center (NHTC), a branch of MOHP for supporting in-service training, is responsible for disseminating approved provider education at its centers throughout the country. Since the official adoption of the HBB by the MOHP, NHTC has orchestrated multiple HBB training-of-trainer (TOT) courses throughout the country in collaboration with Latter-day Saint Charities (LDSC)^16^ and Safa Sunaulo Nepal (SSN). LDSC, the sponsor of these HBB TOT courses, is the humanitarian arm of The Church of Jesus Christ of Latter-day Saints, and SSN is a nongovernmental organization that is the local implementing partner in Nepal.

A major challenge in implementation and dissemination efforts for newborn resuscitation has been maintaining resuscitation skills over time. Several HBB follow-up studies have shown a decline in knowledge and skills over time.^17^^–^^20^ In 2017, realizing that sustained gains in neonatal mortality require effective scale-up and maintenance of skills, SSN designed a package centered on building the capacity of facility-based trainers to scale up and sustain the skills required to manage newborn emergencies in their respective facilities. A single dedicated external mentor assisted the facility-based trainers and monitored the program using key indicators. In this article, we describe the 21-month implementation of the SSN program of scaling up newborn resuscitation training and skill retention in Gandaki Province and report changes in newborn outcomes that occurred during the time of program implementation.

A major challenge in implementation and dissemination efforts for newborn resuscitation has been maintaining resuscitation skills over time.

## METHODS

### Program Setting

The newborn resuscitation and retention program was conducted in Gandaki Province, one of the 7 provinces under the new federal structure in Nepal. An overview of the province is provided in Table 1.

**TABLE 1.** Overview of Gandaki Province, Nepal

Total number of districts	11^a^
Total population	2,403,757^a^
Total number of public hospitals	16^a^
Total number of nonpublic hospitals	52^b^
Neonatal mortality rate	15^c^

^a^Gandaki Province Government, Annual Health Report 2076/77.^21^

^b^Department of Health Services, Ministry of Health and Population, Nepal.^22^

^c^Nepal Demographic and Health Survey 2016.^7^

NHTC selected 12 health facilities (7 of 14 level I hospitals and 5 of 6 level II hospitals) in the province to implement the SSN newborn resuscitation program. Selection criteria included: (1) handling a significant share of the deliveries in Gandaki province and (2) having HBB training needs as identified by NHTC. All the hospitals provided vaginal delivery (normal and assisted), and all except 1 provided cesarean delivery. The labor units in these hospitals were led by physicians and midwives and equipped to provide neonatal resuscitation at birth. Pediatricians led neonatal care units at secondary and tertiary hospitals. In primary hospitals, sick newborns were managed in the pediatric unit, which was led by either medical officers or nurses/midwives. All deliveries occurring in the health facilities during the program period were included in this evaluation and selected for analysis.

### Intervention Overview

The program for scaling up and retaining newborn resuscitation skills was sponsored by LDSC and implemented by SSN from March 2018 to November 2019, which corresponds to Chaitra 2074 to Mangsir 2076 on the Nepali calendar. The program components included: (1) clinical equipment and training materials provided to each facility; (2) facility-level scale-up of resuscitation training to all physicians and nurses/midwives; (3) practice sessions, review meetings, and refresher training; (4) on-site coaching; (5) monitoring of key indicators; and (6) liaisons with administration.

Before implementation of the program, 43 trainers representing the cohort of 12 hospitals attended a 2-day HBB TOT course on April 23–24, 2017. These trainers completed the standard HBB curriculum, combined with additional facilitator training. NHTC directed and supervised the training and led the selection of both facilities and trainers.

Twenty-seven of 43 trainers (63%) were nurses by profession, with the majority (70%) of the trainers working in their profession for more than 5 years. Nearly 40% of the participants were from district hospitals. Twenty-nine trainers (67%) indicated they personally attended up to 25 deliveries in an average month. Thirty-four (79%) trainers stated they resuscitated babies with a bag and mask as part of their current job.

The HBB TOT course was facilitated by 9 Nepali trainers and 4 U.S.-based trainers from LDSC. SSN and LDSC provided trainers with print and electronic copies of instructional materials, such as provider guides and testing materials. Training equipment included NeoNatalie manikins, Penguin suction devices, bag and masks, and stethoscopes. Bag and masks and suction devices were sufficient to set up practice corners and for clinical use in the delivery rooms. LDSC and SSN provided training, replacement instructional materials, and clinical equipment throughout the program as needed and after program completion.

An HBB-trained medical officer with research experience was recruited to serve as a mentor, trainer, monitor of facility-based indicators, and administrative liaison to implement newborn resuscitation scale-up and retention for all the facilities. The mentor was the initial driver for scaling up newborn resuscitation in the selected facilities through the use of evidence-based methods to build staff capacity. Under the mentor’s supervision, the newly minted facility-based trainers conducted HBB training for physicians, nurses, and midwives in the labor and neonatal care units. Training sessions were subsequently provided for new staff due to staff turnover and task-sharing.

The mentor was the initial driver for scaling up newborn resuscitation in the selected facilities through utilization of evidence-based methods to build staff capacity.

After the facility-level scale-up, daily practice sessions were strongly encouraged, using low-dose, high-frequency^23^ bag-and-mask ventilation practice. Other tools for retaining skills included post-resuscitation self-evaluation forms, weekly group review sessions led by the facility-based trainer, monthly resuscitation debriefings led by the mentor, refresher training, and communication regarding goals and progress.

The mentor, in coordination with the facility-based trainer and the hospital nursing supervisor, observed the HBB skills of staff using a neonatal simulator during regular and unannounced site visits; reviewed the practice logbook; provided on-site coaching; reviewed the delivery and newborn logbook; and provided refresher training. Other monitoring activities involved regular phone calls between mentor and trainers, encouragement to maintain standardized delivery room records, and feedback to facility-based trainers based on the review of monthly monitoring reports by SSN representatives. Further, the facility-based trainers communicated with the mentor and each other through dedicated social media groups.

### Evaluation Design

We evaluated the program with a prospective cohort design to compare outcomes of birth cohorts in 12 hospitals following the implementation of the resuscitation training program. All deliveries occurring in the health facilities during the program period were included in the evaluation. The 21-month program followed the Nepali calendar, consistent with the reporting convention of the Nepali government. The program period began in March 2018 (Chaitra 2074) and ended in November 2019 (Kartik 2078).

The primary evaluation outcome included the pre-post difference in (1) the intrapartum/fresh stillbirths and (2) neonatal deaths within the first 24 hours of life. Stillbirths were considered fresh if they were not macerated. Secondary outcomes included neonatal deaths after the first 24 hours of life with hospital discharge as the endpoint and the use of bag-and-mask ventilation on nonbreathing newborns. Secondary outcomes also included the number of sick newborns discharged from the hospital. Sick newborns discharged from the hospital included their transfer from the maternity unit to another unit of the hospital or to another hospital or discharged home before recovery. It was a proxy for all-cause newborn morbidity during the first days of life.

### Data Collection and Management

Monthly monitoring reports generated by the mentor documented the scale-up and facility support as well as facility-level metrics. The monthly scale-up and facility support included tracking the number of providers trained and retrained; maintenance of an equipped practice corner and low-dose, high-frequency practice logs; completion of self-evaluation forms; review of meetings held; and review of delivery and newborn logs. Monthly facility-level metrics included the number of vaginal deliveries, number of cesarean deliveries, number of intrapartum (fresh) and macerated stillbirths, number of neonatal deaths within and after 24 hours, and the number of sick newborns transferred or discharged from the maternity unit.

In private and higher-level public facilities, monthly facility-level data were derived from the health information department, which tabulated and reported MOHP-mandated metrics. In smaller facilities, the mentor gathered data from registers or logs maintained in the labor room and in the maternity ward. Personal data of patients, such as demographic information, were not collected.

### Data Analysis

The monthly scale-up and support measures were largely comprised of qualitative indicators. An account of these is presented in descriptive terms. We used a Poisson regression model to analyze the trend in the adverse birth outcomes following the implementation of the LDSC/SSN program. Clustering for repeated measurements at the facilities was adjusted with generalized estimating equations model specification.

The mentor collected data from standard hospital reports and logs into monthly reports, as well as conducted checks for data quality and integrity. The monthly reports were forwarded to SSN and to a U.S.-based research assistant. SSN conducted an initial review and analysis and prepared MOHP-mandated reports. The data were combined into a master file, after which missing variables were clarified with the mentor, variables missing significant data were deleted, and text was changed into appropriate numerical values. A Nepal-based research assistant conducted the analysis using Excel and SPSS.

### Ethical Approval

Ethical approval was obtained from the Nepal Health Research Council (registration number 797/2018) for the newborn resuscitation scale-up and retention program, including data collection from all facilities under the program.

## RESULTS

### Facility Profile

The evaluation included 12 health facilities, of which 9 were government owned and 3 were nongovernment owned. Government-owned facilities represented 3 types of health service delivery institutions: primary hospital (1), secondary hospital (7), and tertiary hospital (1). Nongovernment health facilities included in the program provided tertiary-level services and were major referral centers in the province.

According to the Health Infrastructure Development Standards 2017, primary hospitals in Nepal are mandated to provide normal delivery and comprehensive emergency obstetric and neonatal care. However, in practice, availability of these latter services in the primary hospital was sporadic, depending upon the availability of physicians. Besides comprehensive emergency obstetric and neonatal care services, both secondary and tertiary hospitals have dedicated departments and wards for providing gynecological and obstetric services. In addition, tertiary hospitals have advanced infrastructures and human resources for providing maternal and newborn care, including a higher number of beds, neonatal care units, and pediatricians. Delivery services were supervised by obstetrician-gynecologists in both secondary and tertiary hospitals.^24^

### Perinatal Outcomes

**FIGURE 1:**
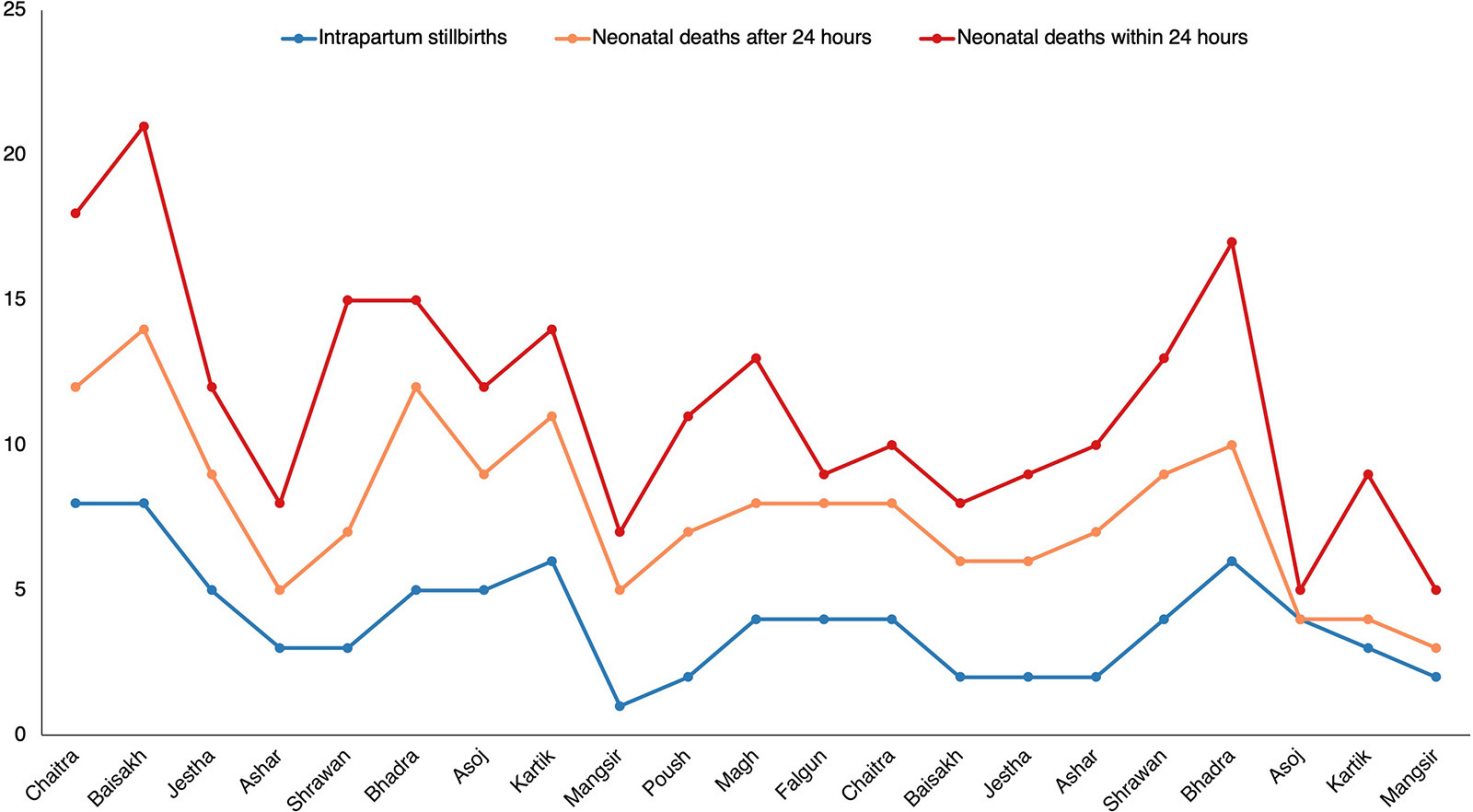
Intrapartum Stillbirths, Neonatal Deaths Within 24 Hours, and Neonatal Deaths After 24 Hours at Program Facilities in Gandaki Province, Nepal, March 2018–November 2019

**FIGURE 2:**
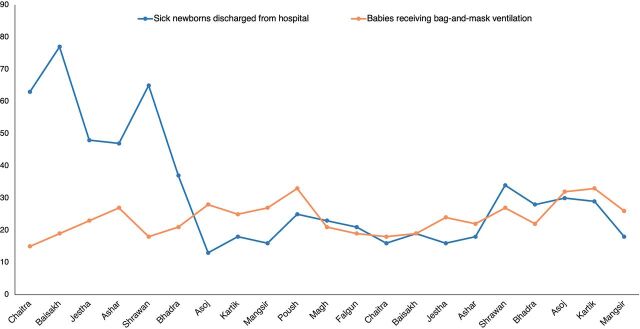
Newborns Receiving Bag-and-Mask Ventilation and Newborn Morbidity at Program Facilities in Gandaki Province, Nepal, March 2018–November 2019

During the program period, a total of 33,417 deliveries took place at the hospitals (mean=128.89, standard deviation=174.85), of which 23,820 were vaginal deliveries and 9,597 were cesarean deliveries. The run chart depicting the trend of intrapartum stillbirth and neonatal mortality within and after the first day of life is shown in Figure 1. Similarly, the trend over time of assisted ventilation performed on nonbreathing newborns and newborn morbidity is presented in Figure 2.

Table 2 summarizes the findings from the Poisson regression on the selected HBB program indicators. Two outcome variables showed a significant association with time following the implementation of the LDSC/SSN capacity-building and skill retention program. Neonatal deaths within 24 hours of birth showed a significantly declining trend (incidence rate ratio=0.993, *P*=.044), a 0.7% decrease in neonatal mortality for every unit change in time. The number of sick newborns transferred from the hospital was significantly associated with time (incidence rate ratio=0.996, *P*<.001), revealing a 0.4% reduction in the number of sick newborns transferred from the hospital for every unit change in time.

**TABLE 2.** Trend Analysis of Newborn Resuscitation Capacity Building and Skill Retention Program Outcomes, Gandaki Province, Nepal

**Outcomes**	**Covariate (Time)**	
	**Incidence Rate Ratio**	***P* Value**
Intrapartum stillbirths	1.000	.569
Within 24 hours neonatal mortality	0.993	.044
After 24 hours neonatal mortality	1.130	.412
Bag-and-mask ventilation performed on nonbreathing newborns	1.000	.104
Number of sick newborns discharged from the hospital	0.996	<.001

### HBB Scale-Up and Support

**FIGURE 3:**
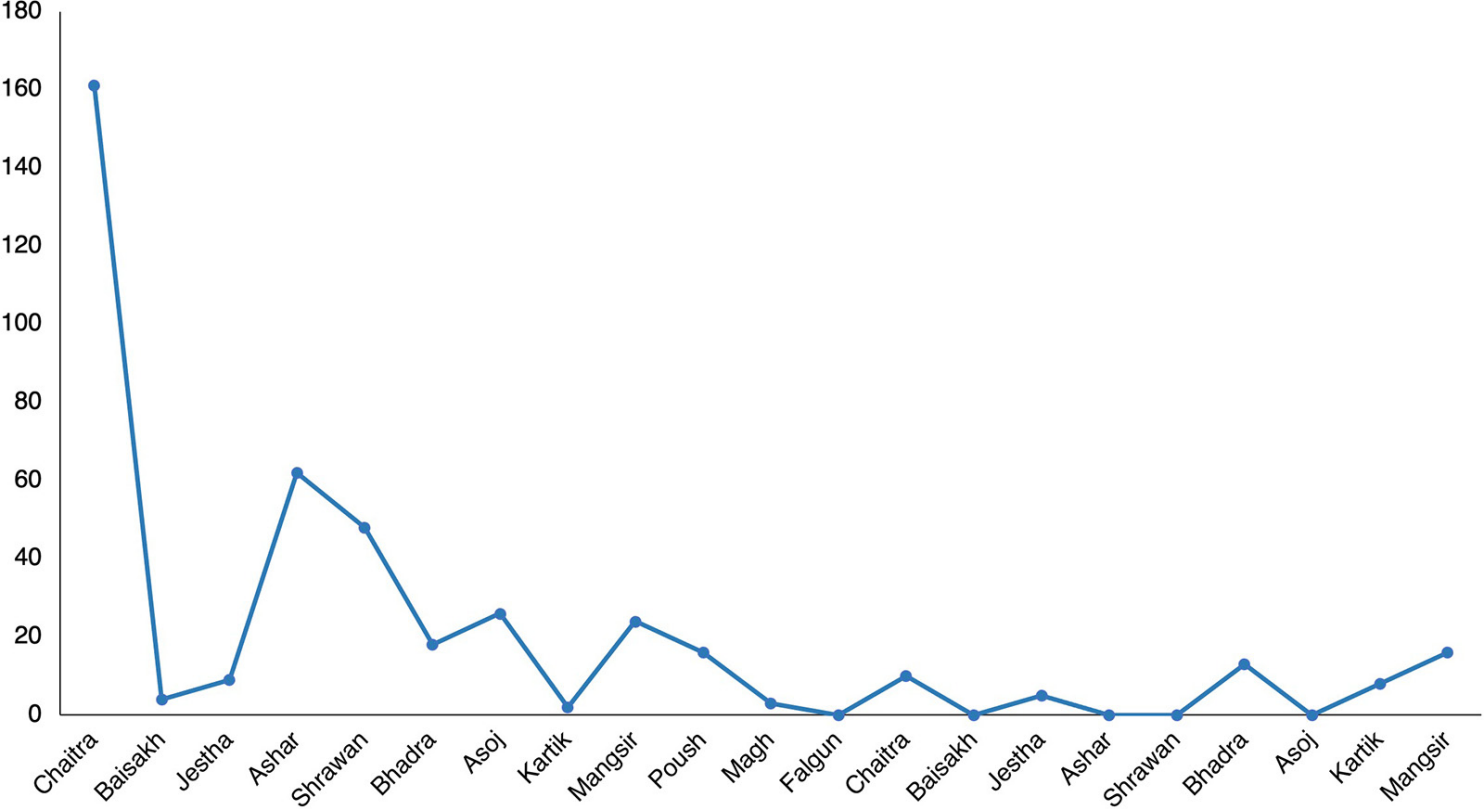
Number of Facility-Based Providers Trained With Bag and Mask at Program Facilities in Gandaki Province, Nepal, March 2018–November 2019

The scale-up of HBB resulted in 425 providers receiving training during the program period. The new facility-based trainers, with the assistance of the mentor, cascaded the training in their facilities by training existing and new staff during the program and holding refresher training sessions. Most of the initial training of staff (41%) took place in the first 3 months (Figure 3), while only 5% were trained in the last 3 months. In the final months of the program, training of new staff continued while training was expanded to include nonpermanent staff such as simulation-based assessment trainees, nursing students, and residents/students not aligned with program hospitals.

The new facility-based trainers, with the assistance of the mentor, cascaded the training in their facilities by training existing and new staff during the program and holding refresher training sessions.

A detailed review of data contained in the monthly reports revealed consistent attendance by the mentor at resuscitation reviews with staff during the entire program period, averaging 1.4 meetings per month per hospital. Frequent meetings with nursing supervisors, medical superintendents, and other hospital leaders were also consistent throughout the program but with a higher frequency during the first half of the period, averaging at least once every 2 months. The mentor assisted the facility-based trainers both with consistent practice sessions and review discussions with available staff and with training of new staff. In contrast, the utilization of self-evaluation surveys was poor, and practice corners with completion of practice logs were only scaled up to two-thirds of the hospitals by the end of the program. Practice sessions of staff with the mentor or facility-based trainer were more consistent and universal among the facilities than spontaneous practice by providers. All units remained fully equipped during the program, and LDSC provided replacement equipment as needed. All units maintained both maternal and newborn logs during the entire program, though coaching by the mentor on proper completion of the logs was needed.

## DISCUSSION

The SSN program was implemented in a phase-wise approach in 5 provinces of Nepal. It was implemented first in Western Nepal and subsequently in Gandaki province. The hospitals in this evaluation were in a significantly more developed region in terms of socioeconomic status, access to health care, and hospital infrastructure than in Western Nepal. Different external mentors were recruited in various locations. A significant association of the program with neonatal mortality within 24 hours of birth as we found in this evaluation was also reported after the implementation of the program in Western Nepal.^25^ We also found that the number of sick newborns transferred from the hospital showed a declining trend (0.4% each month, *P*<.001). Our findings correspond with several previous studies that found significant decreases in neonatal mortality within 24 hours of birth^13^^,^^26^^,^^27^ following the HBB training. Overall, this evaluation validates the effectiveness of the SSN program in scaling up newborn resuscitation training and skill retention in improving perinatal outcomes in Nepal.

Our findings correspond with several previous studies that found significant decreases in neonatal mortality before 24 hours of birth following the HBB training.

Analysis of the run charts indicates fluctuations in the number of deaths within and after 24 hours of birth and in intrapartum stillbirths over the period of program implementation. On the other hand, the number of babies receiving bag-and-mask ventilation increased gradually over time, suggesting a transfer of skills into the clinical setting. Since the program used minimal MOHP-mandated metrics for program monitoring, data collection procedures for neonatal mortality within and after 24 hours of births and fresh stillbirths were already established at the onset of the SSN’s program implementation. However, data collection procedures for data on newborn morbidity, use of bag and mask, facility-level scale-up, and skill retention metrics were initiated at the onset of program implementation. This may have affected the data quality in the early period of the program.

Findings from our evaluation revealed a proportionally higher percentage of cesarean deliveries (28.7%). Of 12 health facilities included in the program, 7 were advanced-level facilities where complicated obstetric cases were referred. Thus, the increased need for cesarean deliveries to manage obstetric complications in these health facilities may have resulted in higher percentages of cesarean deliveries. These facilities accounted for nearly 92% (data not shown) of the cesarean deliveries in the program hospitals.

The rollout of the newborn resuscitation capacity-building and skill retention program at scale by SSN utilized quality improvement strategies from earlier studies to sustain newly acquired resuscitation skills. A clinical trial in a tertiary-level hospital in 2013 in Nepal used the quality improvement process cycle to demonstrate HBB’s impact on perinatal outcomes.^17^ These included HBB training, daily practice sessions, post-resuscitation self-evaluation, and weekly review meetings. In addition, the current initiative focused on the scaling up of newborn resuscitation capacity and skill retention through facility-based cascade training and frequent refresher trainings by hospital-based trainers. The program also emphasized the catalytic role of the mentor in capacity-building and skill retention through collaboration with hospital clinical leadership in establishing systems for ongoing practice, mentorship, supervision, and institutionalization of newborn resuscitation skills. Toward the conclusion of the program, most of the hospitals had transitioned to the appointment of an internal mentor for sustainability.

Several recommendations emerge from the review of mentor functions, some of which are consistent with earlier studies but some of which are not. Based on the scale-up experience in Gandaki, a similar program would need at least 6 months to scale up training and institute retention activities in a portfolio of health care facilities. The role of the mentor was critical in training and assisting the hospital-based facilitators during this time, including holding regular meetings with staff, liaising and advocating with hospital leaders, conducting ad hoc practice sessions and discussions, ensuring the training of new staff, and improving the accuracy of log-based documentation. The establishment and spontaneous staff utilization of practice corners had limited success, and post-resuscitation self-evaluation survey completion was widely unsuccessful. Successful transition to an internal mentor depends highly on support from hospital administration, which in turn needs support from local health authorities.

The goal of scale-up is to gain effective coverage of the intervention and institutionalize the intervention as a routine practice within the national health system.^28^ Scaling up interventions at the population level is complex, and there is no single pathway to accomplish it.^29^ Despite evidence suggesting effectiveness of the HBB approach in reducing perinatal outcomes, very few countries have scaled up HBB nationwide. SSN’s program could serve as a model to guide the scale-up of newborn resuscitation capacity-building and skill retention in low-resource settings. For example, the Nepalese MOHP could adopt the mentorship model by appointing an internal mentor in health facilities with external oversight by local health departments. An internal mentoring and external supervision approach could be efficient in overcoming geographical barriers and pervasive human resource constraints in the Nepalese health system.

### Strengths

A notable strength of the program has been the dedicated commitment and leadership of NHTC, under the MOHP, in conducting HBB training. Beginning before the Gandaki TOT course, SSN received guidance and coordination support from NHTC and the MOHP regarding HBB TOT courses. This support included development and endorsement of an HBB learning resource package (based on the American Academy of Pediatrics module), identification of facilities and human resources requiring HBB training through pretraining needs assessment, technical assistance in training sessions, joint monitoring/supervision of HBB TOT sessions and refresher trainings, recordkeeping of the training equipment provided to the health institutions during HBB TOTs, and strategic direction for effective program implementation. This clearly demonstrates the role of country ownership and leadership in successfully implementing newborn resuscitation skills at scale. The role of a dedicated mentor in scale-up and skill retention was also crucial. These observations are not unique to the SSN’s program of scale-up but have been acknowledged in the scale-up of various maternal and child health interventions.^29^

The SSN program was a relatively low-cost intervention. A single external mentor design allows diffusion of associated costs among health facilities. The facility-level cascade training approach incurred a low direct cost to SSN by avoiding travel costs and daily subsistence allowance, compared to off-site training. Indirect costs associated with loss of work hours were also markedly reduced as the facility-level providers were trained while they were still on the job.

The SSN program was a relatively low-cost intervention because of its single external mentor design, facility-level cascade training approach, and use of on-the-job training.

Another strength of the program is that the participating facilities constituted a large proportion of deliveries in the Gandaki province. Further, the facilities included were diverse, ranging from district-level public hospitals to tertiary-level public and private hospitals providing advanced maternal and newborn services.

### Challenges

The program faced several challenges. First, many facilities had a low staffing ratio, leading to unavailability of staff for the facility-based training. Second, widespread staff turnover across facilities, particularly during the human resource adjustments by the Government of Nepal toward the end of the program period, led to the transfer of skilled human resources to other areas and an influx of untrained staff into the facilities. This not only affected the overall quality of newborn care but also posed data collection challenges.

The third challenge related to the data collection process itself. The program monitored progress by using limited indicators, most of which were tracked by the facilities for routine reporting to the MOHP and, therefore less prone to error—neonatal deaths being one of those metrics. However, even though most of the facilities tracked stillbirths, misclassification and underreporting of fresh stillbirth was widespread. Accordingly, delivery logs had to be reviewed to confirm accuracy in classification of fresh stillbirth. Similarly, since the facilities did not track and report resuscitation data to the MOHP, delivery logs had to be reviewed to obtain such data. Sick newborns discharged from the maternity unit—a proxy indicator for all-cause morbidity—were also not tracked by facilities as part of routine reporting to the MOHP. This necessitated a review of newborn logs to determine the number of sick infants discharged from the units.

Due to the design as a skill training and retention program rather than a clinical study, there was no control/comparison group, which restricted the ability to establish the causality of the program on observed perinatal outcomes. We describe the impact of the newborn resuscitation scale-up on perinatal outcomes using routinely collected data, without establishing the causality, as in clinical trials. With this, we are unable to confirm whether the observed outcomes were solely attributable to the newborn resuscitation scale-up and skills retention program or to other changes at the facilities during the period.

Various studies have documented seasonality of adverse birth outcomes in Nepal. Seasonal variation in neonatal deaths (highest risk in August and lowest risk in March) and stillbirths (highest risk in February and lowest in October) was observed in a randomized control trial conducted in rural Nepal from 2002 to 2006.^30^ A recent cluster-randomized controlled trial conducted in the southern plain region of Nepal also noted a seasonal pattern in neonatal size and maternal nutrition with the highest risk of adverse neonatal anthropometry in the hot season (May/June).^31^ The seasonal trends in adverse birth outcomes as established by these studies might have affected the occurrence of perinatal outcomes under this evaluation and could have biased the estimates of association observed in this evaluation. Hence, we recommend that the findings from this report be interpreted and generalized with caution.

HBB refresher training was conducted in one of the health facilities by UNICEF during the implementation period, augmenting the scale-up of training but not contributing to the retention of skills portion of the SSN program. Likewise, the MOHP training on Community-Based Integrated Management of Childhood Illness and the Skilled Birth Attendant In-service Training Packages provided to some facility staff by the Government of Nepal used the basic HBB algorithm but without the practice components usually deemed necessary for skill acquisition. With these and other types of newborn interventions possibly running concurrent to the SSN program, the exact contribution of the SSN model of newborn resuscitation scale-up and retention program to the observed improvements in neonatal outcomes is difficult to quantify.

Interestingly, nearly 79% of trainers in the TOT were already resuscitating babies in their current job at the time of training. Yet, neonatal outcomes improved despite most of the trainers having prior knowledge and experience with neonatal resuscitation. This emphasizes that training alone is insufficient to achieve gains in neonatal outcomes and needs to be followed by practice, mentorship, retraining, and monitoring—the main focus of our efforts.

Training alone is insufficient to achieve gains in neonatal outcomes and needs to be followed by practice, mentorship, retraining, and monitoring.

This report highlights the potential of the SSN program of newborn resuscitation scale-up and retention to improve perinatal outcomes in low-resource settings through building and sustaining in-facility capacity for resuscitation. Minimal resources were utilized to accomplish this. The report also highlights the complexity of implementing and scaling up evidence-based newborn resuscitation interventions.

## CONCLUSION

Our evaluation provides plausible evidence that the SSN program of scaling up newborn resuscitation and skill retention was associated with a decline in neonatal mortality within 24 hours, a decline in the number of sick newborns transferred from the maternity unit, and an improvement in clinical practices. This evidence-based approach is a simple program focused on facility-based cascade training of newborn resuscitation skills using the HBB curriculum, followed by practice, mentorship, and other skill retention strategies. The program demonstrated the role of mentorship in effectively scaling up skills, retaining skills, and monitoring, with a single mentor supervising and monitoring multiple facilities. The program could guide future programs to reduce perinatal outcomes for other resource-limited countries striving to meet the Sustainable Development Goal target of reducing neonatal mortality to at least 12 per 1,000 live births.

## Notes

**Peer Reviewed**

**First published online:** February 14, 2023.
